# Four New Sequence Types and Molecular Characteristics of Multidrug-Resistant *Escherichia coli* Strains from Foods in Thailand

**DOI:** 10.3390/antibiotics13100935

**Published:** 2024-10-02

**Authors:** Nalumon Thadtapong, Soraya Chaturongakul, Sithichoke Tangphatsornruang, Chutima Sonthirod, Natharin Ngamwongsatit, Ratchaneewan Aunpad

**Affiliations:** 1 Graduate Program in Biomedical Sciences, Faculty of Allied Health Sciences, Thammasat University, Pathum Thani 12121, Thailand; nalumonth.thadtapong@gmail.com; 2 Center for Advanced Therapeutics, Institute of Molecular Biosciences, Mahidol University, Nakhon Pathom 73170, Thailand; soraya.cha@mahidol.ac.th; 3 Pornchai Matangkasombut Center for Microbial Genomics (CENMIG), Faculty of Science, Mahidol University, Bangkok 10400, Thailand; 4 National Center for Genetic Engineering and Biotechnology, National Science and Technology Development Agency (NSTDA), Pathum Thani 12120, Thailand; sithichoke.tan@biotec.or.th (S.T.); chutima.son@biotec.or.th (C.S.); 5 Department of Clinical Sciences and Public Health, Faculty of Veterinary Science, Mahidol University, Nakhon Pathom 73170, Thailand; natharin.nga@mahidol.edu; 6 Laboratory of Bacteria, Veterinary Diagnostic Center, Faculty of Veterinary Science, Mahidol University, Nakhon Pathom 73170, Thailand

**Keywords:** *Escherichia coli*, food safety, phenotype–genotype correlation, whole genome sequencing, epidemic

## Abstract

The presence of antibiotic-resistant *Escherichia coli* in food is a serious and persistent problem worldwide. In this study, 68 *E. coli* strains isolated from Thai food samples were characterized. Based on antibiotic susceptibility assays, 31 of these isolates (45.59%) showed multiple antibiotic resistance (MAR) index values > 0.2, indicating high exposure to antibiotics. Among these, strain CM24E showed the highest resistance (it was resistant to ten antibiotics, including colistin and imipenem). Based on genome sequencing, we identified four isolates (namely, CF25E, EF37E, NM10E1, and SF50E) with novel Achtman-scheme multi-locus sequence types (STs) (ST14859, ST14866, ST14753, and ST14869, respectively). Clermont phylogrouping was used to subtype the 68 researched isolates into five Clermont types, mainly A (51.47%) and B1 (41.18%). The *bla*_EC_ gene was found only in Clermont type A, while the *bla*_EC-13_ gene was predominant in Clermont type B1. A correlation between genotypes and phenotypes was found only in Clermont type B1, which showed a strong positive correlation between the presence of an *afa* operon and yersiniabactin-producing gene clusters with the colistin resistance phenotype. Strain SM47E1, of Clermont type B2, carried the highest number of predicted virulence genes. In summary, this study demonstrates the pressing problems posed by the prevalence and potential transmission of antimicrobial resistance and virulence genes in the food matrix.

## 1. Introduction

The *Enterobacteriaceae* family includes several problematic Gram-negative bacteria recognized for their role in disease etiology and antimicrobial resistance, and *Escherichia coli* is one of the key members in this family [1]. *E. coli* inhabits the gastrointestinal tracts of animals and humans [2]; it is also a fecal coliform organism and is therefore used as an indicator of good hygiene and food safety assessment [3,4]. Colistin is a last-resort drug for treating the invasive infections caused by antibiotic-resistant *E. coli* [5]; however, colistin-resistant *E. coli* was identified in 2015 [6]. The *mcr* gene confers colistin resistance, and *mcr*-mediated colistin-resistant *E. coli* is known to have spread worldwide [7,8]. The ability of *E. coli* to acquire and produce extended-spectrum beta-lactamase (ESBL) has led the bacterium to be recognized as a “Critical tier” global priority pathogen by the World Health Organization (WHO) [9].

In Thailand, an analysis of the assessed health burden due to antimicrobial-resistant (AMR) bacteria from 2009 to 2010 showed that at least 90,000 patients per year were hospitalized with antimicrobial-resistant bacterial infections; approximately one-third of them died [10], and the total cost of treatment for AMR infections rose to USD 1.3 billion [10,11]. Recently, the National Antimicrobial Resistance Surveillance Center, Thailand (NARST), reported that in 2021, data from 51 hospitals confirmed that ESBL-producing *E. coli* was 46.8% resistant to cefotaxime and 38.2% resistant to ceftazidime [12]. *E. coli* contamination in food and drinking water is a major cause of foodborne illness in humans [1,3]; thus, risk assessments of *E. coli* prevalence, colistin resistance, and ESBL-producing profiles are continually needed [13].

*E. coli* isolated from foods in Thailand showed a high prevalence of antibiotic resistance, including ESBL producers. In 2014, *E. coli* and ESBL-producing *E. coli* were found in 40% and 33.3% of fresh pork meat from slaughterhouses in a northern province and an eastern province, respectively [11]. In Bangkok and the central provinces, *E. coli* and ESBL-producing *E. coli* were isolated in almost 90% and 80% of fresh food samples from markets, respectively [11]. In 2016, the prevalence of *E. coli* from swine and broiler slaughterhouses was 36–85% in Sa Keao province, 77.4% of which were multidrug-resistant (MDR) *E. coli* [14].

Our recent report indicated that *E. coli* contamination was detected in 72% of sample food specimens (raw and fermented meats) using multiplex PCR and culture methods [15]. Even though *E. coli* O157:H7 was not identified among the isolated strains, antibiotic resistance and other virulence factors involved in the pathogenicity of *E. coli* are still unknown [15]. In order to further investigate and monitor the incidence of antibiotic resistance and virulence among these strains, the current study was focused on the detection of colistin-resistant and ESBL-producing *E. coli*, as well as genomic analyses for the identification of antibiotic resistance, virulence gene patterns, and plasmids. The correlation between phenotype (antibiotic resistance) and genotype (antibiotic resistance and virulence genes) was also explored. The resulting information provided insights into the current situation of antibiotic-resistant and virulent *E. coli* distribution in Thailand, as well as its mobile genetic elements. In this study, we found that 30 isolates (44.12%) were MDR *E. coli*, while seven isolates showed resistance to last-line drugs. We also identified four strains with novel sequence types (STs) and observed patterns of *bla* and virulence genes associated with Clermont types. The plasmid replicons detected in the genomic data exhibited high diversity, and our findings indicate that *E. coli* isolates from contaminated food in Thailand possess a variety of genotypes and high-risk virulent clones, information that is useful for the future surveillance of antibiotic resistance and virulence gene transmission among *E. coli* in the food chain.

## 2. Results

### 2.1. Prevalence of Antibiotic-Resistant E. coli

**Figure 1:**
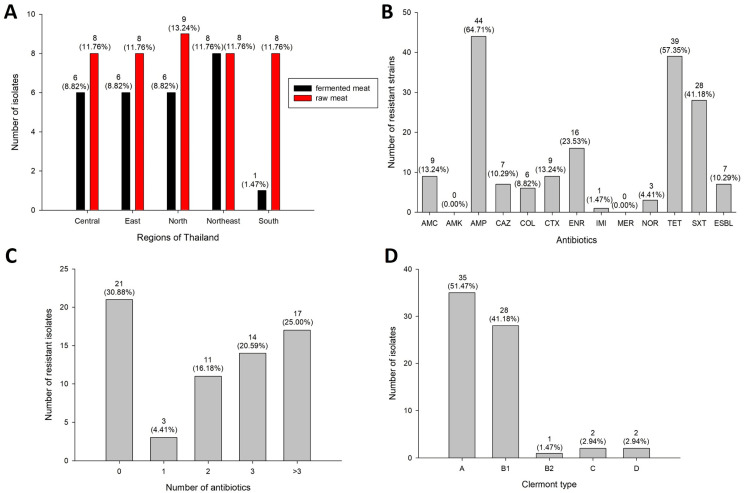
Distribution of isolates and AMR patterns of *E. coli* used in this study. (**A**) Prevalence of *E. coli* in fermented meat and raw meat from five regions of Thailand. Black and red bars represent the sources of isolates, fermented meat and raw meat, respectively. (**B**) Prevalence of single antibiotic resistance. Distribution of (**C**) multidrug resistance and (**D**) the four Clermont types of *E. coli* isolates. Numbers of isolates or resistant isolates are shown at the top of each bar, with the percentages of isolates represented in parentheses. AMC, amoxicillin–clavulanic acid; AMK, amikacin; AMP, ampicillin; CAZ, ceftazidime; COL, colistin; CTX, cefotaxime; ENR, enrofloxacin; IMI, imipenem; MER, meropenem; NOR, norfloxacin; TET, tetracycline; SXT, sulfamethoxazole–trimethoprim; ESBL, extended-spectrum beta-lactamase-producing.

Sixty-eight *E. coli* strains were isolated from raw and fermented meats from five different regions of Thailand [15]. The prevalence of isolated *E. coli* was revealed and is illustrated in Figure 1A. Antibiotic susceptibility testing was conducted for 12 antibiotics using disk diffusion and microdilution (Figure 1B and Supplemental Table S1). No *E. coli* isolates were resistant to amikacin (AMK) or meropenem (MEM), while twenty-one isolates (30.88%) were susceptible to all 12 antibiotics (Figure 1C and Table 1). Most *E. coli* isolates showed ampicillin (AMP) resistance (n = 44/68, 64.71%), thirty isolates (44.12%) were MDR (i.e., resistant to three or more antibiotic classes [16]) strains, eleven isolates (16.18%) were ampicillin–tetracycline–sulfamethoxazole–trimethoprim (AMP-TET-SXT)-resistant strains, and thirteen more MDR isolates (19.12%) exhibited at least this pattern. For last-line drugs, six isolates (8.82%; CM24E, EM39E1, NEM17E1, NEM20E, NM1E1, and SM48E) and one isolate (1.47%; CM24E) showed colistin (COL) and imipenem (IMI) resistance, respectively. Isolates that resist cefotaxime (CTX) (n = 9/68, 13.24%) and ceftazidime (CAZ) (n = 7/68, 10.29%) were selected to test ESBL production using a phenotypic confirmatory disk diffusion test (PCDDT). Seven isolates (10.29%) were ESBL-producing strains. In order to accumulate MDR capabilities, these AMR *E. coli* isolates had to be exposed to a series of antimicrobials. The MAR index was then calculated for each strain (Table 1), and values greater than 0.2 indicated that the isolates were from high-risk sources with heavy antibiotic use; thirty-one isolates (45.59%) showed MAR index values > 0.2.

### 2.2. New STs and Prevalence of Clermont Types A and B1 in Thailand

**Figure 2:**
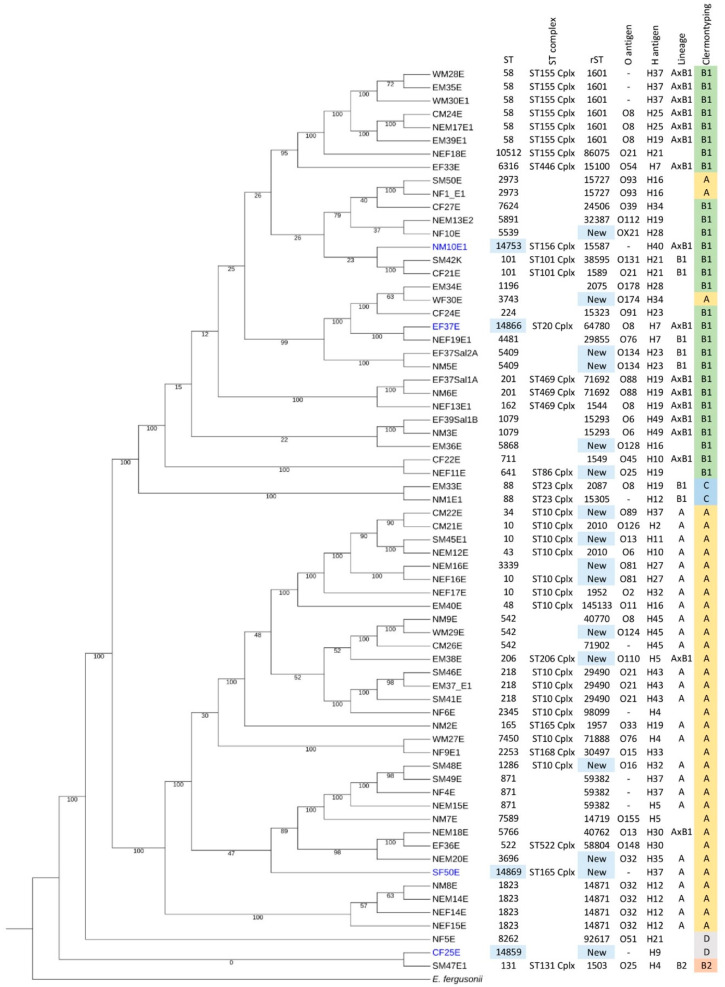
Core genome phylogenetic tree of 68 *E. coli* isolates. Results of the phylogenetic tree are combined with identification data, including ST, ST complex, rST, O antigen, H antigen, lineage, and Clermont type. Four isolates with new STs are highlighted in blue. New STs and rSTs are highlighted and labeled “New”. Clermont types A, B1, B2, C, and D are highlighted in yellow, green, orange, blue, and gray, respectively. *E. fergusonii* NCTC 12128 was used as the outgroup for analysis.

In order to characterize these *E. coli* isolates, whole genome sequencing and analyses were performed. Data from the quality assessment of assembled genome sequences in *E. coli* isolates are presented in Supplemental Table S2. Sequence typing (ST) and ribosomal sequence typing (rST) were analyzed based on the Achtman scheme (Supplemental Table S3) and ribosomal genes, respectively. ST58 (n = 6/68, 8.82%) and ST10 complex (n = 13/68, 19.12%) were the most prevalent ST and ST complex, respectively. We reported four isolates (CF25E, EF37E, NM10E1, and SF50E) with new STs (ST14859, ST14866, ST14753, and ST14869) and 16 isolates (CF25E, CM22E, EF37Sal2A, EM36E, EM38E, NEF11E, NEF16E, NEM16E, NEM20E, NF10E, NM5E, SF50E, SM45E1, SM48E, WF30E, and WM29E) with new rSTs, as confirmed by the creation of new ST numbers in Enterobase. Clermontyping of *E. coli* was also confirmed in Enterobase. We found that the majority of our isolates were in Clermont type A (n = 35/68, 51.47%), followed by type B1 (n = 28/68, 41.18%), and types C and D, with two isolates (2.94%) each (Figure 1D). In Figure 2, two phylogroup types are represented in the core genome phylogenetic tree: the lineage phylogroup, based on Achtman MLST, and the phylogroup based on Clermontyping. Lineage phylogroup A (n = 26/68, 38.23%) and Clermont type A (n = 35/68, 51.47%) were the major phylogroup types identified in our data (Figure 2). The four new STs were distributed among the three following Clermont types: A (SF50E), B1 (EF37E and NM10E1), and D (CF25E). Based on Clermontyping, types A, B1, and C were major clusters for nonpathogenic strains, while types B2 and D were the main clusters for pathogenic strains [17], indicating that most of the isolates were nonpathogenic *E. coli* strains. However, we also found Clermont types B2 (n = 1/68, 1.47%) and D (n = 2/68, 2.94%), with three associated strains that are food isolates with potential in disease etiology.

### 2.3. Clermont Type-Specific bla_EC_ Genes and bla_EC-13_ Presence in ESBL-Producing Strains

**Figure 3:**
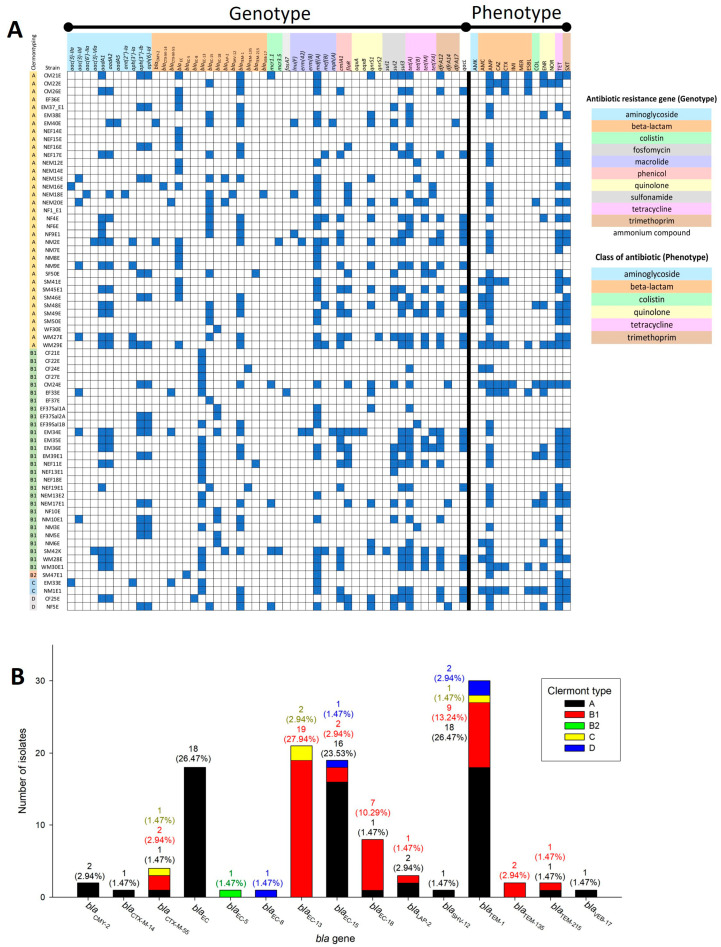
(**A**) Histogram of AMR patterns of *E. coli* isolates based on genotypic and phenotypic data. Blue boxes represent the presence of AMR genes under genotype and resistance under phenotype. Different highlight colors for AMR genes and antibiotics represent different classes of AMR. (**B**) Distribution of *bla* genes in *E. coli* isolates. Clermont types A, B1, B2, C, and D for each *bla* gene is indicated with black, red, green, yellow, and blue, respectively. Numbers of isolates or resistant isolates are shown on top of each bar, with percentages of isolates represented in parentheses. AMK, amikacin; AMC, amoxicillin–clavulanic acid; AMP, ampicillin; CAZ, ceftazidime; COL, colistin; CTX, cefotaxime; ENR, enrofloxacin; IMI, imipenem; MER, meropenem; NOR, norfloxacin; TET, tetracycline; SXT, sulfamethoxazole–trimethoprim; ESBL, extended-spectrum beta-lactamase-producing.

Based on AMR patterns, MDR, last-line drug (COL and IMI) resistance, and ESBL-producing isolates were found in our collection. To investigate the genotypic characteristics of these isolates, genomic data were annotated and predicted for AMR genes using the ResFinder and NDARO databases (Supplemental Table S4). A comparison between AMR genotypes and phenotypes is shown in Figure 3A. Six strains carried *mcr* genes, *mcr1.1* (CM21E, CM24E, NEM17E1, NF5E, and SM42K) and *mcr3.5* (NM2E and SM42K). However, the COL-resistant strains that harbored *mcr* genes were CM24E and NEM17E1. Other COL-resistant strains (EM39E1, NEM20E, NM1E1, and SM48E) did not carry *mcr* genes. In their cases, other mechanisms (e.g., lipid A modification, lipopolysaccharide modification, decrease in negative net charge on the cell surface, loss of lipid A, efflux pump, or outer membrane remodeling [18]), rather than the presence of an *mcr* gene, might confer COL resistance phenotypes.

The beta-lactam (amoxicillin–clavulanic acid, AMC, AMP, CAZ, CTX, or IMI) resistance phenotype comprised the majority of resistance patterns (n = 44/68, 64.71%). Seven ESBL-producing strains (8.82%) contained beta-lactamase (*bla*) genes that play roles in beta-lactam resistance and ESBL production. In the prediction of *bla* genes, we found 15 types of *bla* genes among all 68 isolates (Figure 3B). The *bla*_TEM-1_ gene was the highest prevalent *bla* gene in our collection, found in 30 isolates belonging to four different Clermont types, A (n = 18/68, 26.47%), B1 (n = 9/68, 13.24%), C (n = 1/68, 1.47%), and D (n = 2/68, 2.94%). Interestingly, members of the *bla*_EC_ family were found in all isolates, and some were Clermont type-specific (Figure 3B. Two types of *bla*_EC_ genes, *bla*_EC_ and *bla*_EC-5_, were specific to Clermont types A and B2, respectively. In addition, *bla*_EC-13_, found in Clermont types B1 and C, was not found in Clermont type A. In ESBL-producing strains, we found five patterns of *bla* genes: *bla*_EC_ and *bla*_TEM-1_ (CM21E, CM26E, and WM29E), *bla*_EC-15_ and *bla*_TEM-1_ (CM22E), *bla*_CTX-M-55_ and *bla*_EC-13_ (EF33E), *bla*_EC-13_ (WM30E1), and *bla*_EC-13_ and *bla*_TEM-1_ (NM1E1). Based on Clermont types, ESBL-producing strains in Clermont types A (CM21E, CM22E, CM26E, and WM29E) and B1 (EF33E and WM30E1) harbored *bla*_TEM-1_ and *bla*_EC-13_, respectively, while one strain from type C (NM1E1) harbored both *bla*_TEM-1_ and *bla*_EC-13_. Four common *bla* genes associated with ESBL production among the *Enterobacteriaceae* family were *bla*_CTX-M_, *bla*_TEM_, *bla*_SHV_, and *bla*_OXA_ [19]. The *bla*_CTX-M-55_ gene, which is related to ESBL production [20], was also found in the ESBL-producing strain EF33E.

In our collection, we found only one strain with carbapenem resistance, CM24E, which harbored *bla*_TEM-1_ and *bla*_EC-13_ genes. In general, *bla*_KPC_ (Ambler class A), *bla*_NMD_ (Ambler class B), and *bla*_OXA_ (Ambler class D) are commonly found in carbapenem-resistant *E. coli* [21]. The *bla* from Ambler class C is not considered a carbapenemase gene because of its low potential for hydrolyzing carbapenem and the need for overexpression of an efflux pump or reduced membrane permeability [22]. The *bla*_TEM-1_ and *bla*_EC-13_ genes belong to Ambler classes A and C, respectively [23]. In general, *bla*_TEM_ is associated with resistance to ampicillin, penicillin, and first-generation cephalosporin [24], as well as, in some cases, ESBL production [25]. Hence, CM24E might require an efflux pump and/or low membrane permeability to be associated with the IMI resistance phenotype.

### 2.4. Virulence Patterns in E. coli Isolates and Most Predicted Virulence Genes Found in SM47E1

**Figure 4:**
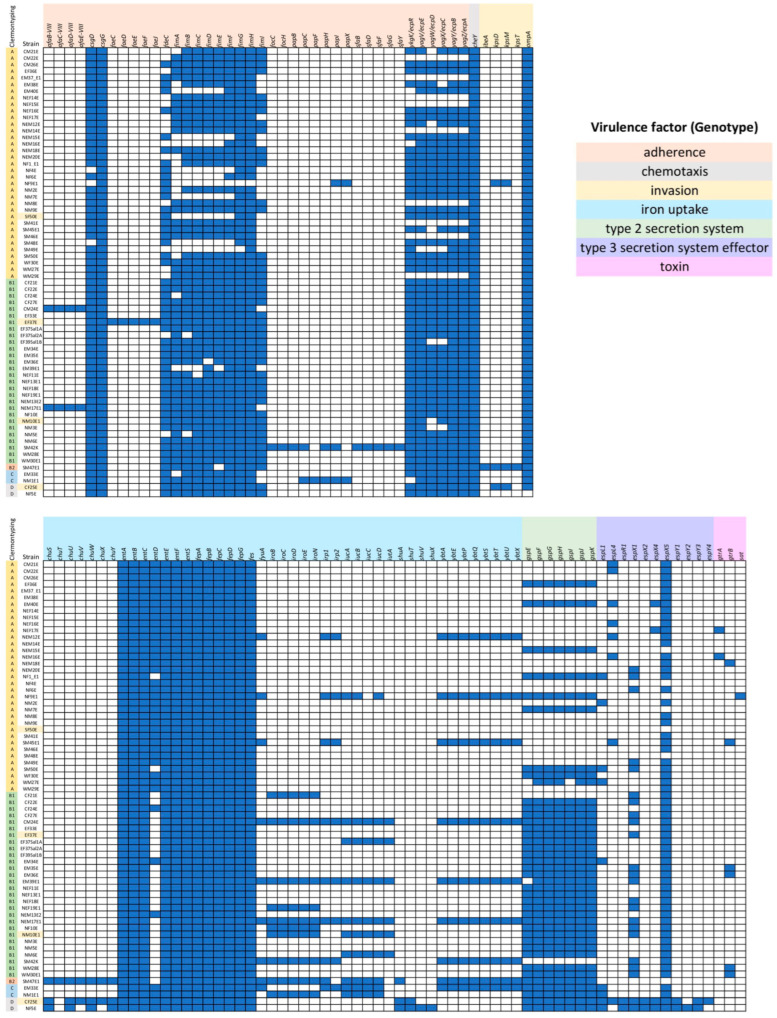
Histograms of virulence gene patterns in the genotypes of *E. coli* isolates. Blue boxes represent the presence of corresponding virulence genes.

Virulence properties in *E. coli* isolates were predicted via genomic analysis, based on the VFDB database (Supplemental Table S5), and the virulence gene patterns are shown in Figure 4. All strains harbored *csgG* (adherence [26]), *cheY* (chemotaxis [27]), *entABCEFS* (*ent* operon, iron uptake [28]), *fepABCDG* (*fep* operon, iron uptake [28]), and *fes* (iron uptake [28]). Some virulence genes were prevalent or lost in specific Clermont types; for example, the gene cluster for the type 3 secretion system (T3SS) effector was found in only Clermont type D (n = 2/2, 100%) and was absent in Clermont type B2 (n = 1/1, 100%). Clusters of *ecpRABCDE* (*ecp* operon for *E. coli* common pilus [ECP] production and adherence [29], n = 25/28, 89.29% of Clermont type B1), *fimABCDEFGH* (*fim* operon for type 1 pilus production and adherence [30], n = 20/28, 71.43% of Clermont type B1), and *gspEFGHIJK* (*gsp* operon, type 2 secretion system (T2SS) [31], n = 26/28, 92.88% of Clermont type B1) were highly prevalent in Clermont type B1. The *gsp* operon was also found in Clermont type B2 (n = 1/1, 100%), C (n = 2/2, 100%), and D (n = 2/2, 100%). The general core *gsp* operon consisted of the *gspCDEFGHIJKLMNO* combination, along with the minor *gspAB* operon and the independent *gspS*, which encodes T2SS and plays the role of a heat-labile enterotoxin (LT) in enterotoxigenic *E. coli* (ETEC) [32]. However, the absence of active GspD abolishes enzyme secretion [33]. The *gsp* operon in our isolates lacked *gspD*; as a result, we can assume non-functional T2SS and, hence, a low to nonpathogenic potential in Clermont type B1 *E. coli*.

Five genes in the invasion category (*ibeA*, *kpsD*, *kpsM*, *kpsT*, and *ompA*), and the *chuSTUVWXY* (*chu* operon, iron uptake [34]) cluster, were found in SM47E1, which is classified as Clermont type B2. The *ibeA* gene encodes the invasion of brain endothelium protein A, which is involved in the adherent-invasive *E. coli* (AIEC) invasion process [35]. *kpsDMT* genes encode group 3 capsule polysaccharide, which is required for capsule polysaccharide transport across the cytoplasmic membrane to the cell surface [36]. The *ompA* gene encodes outer membrane protein A, which plays a role in maintaining the integrity of the outer membrane and in bacterial conjugation [37]. The *chu* operon encodes heme transport for the uptake of iron from a mammalian host and can be found in AIEC and enterohemorrhagic *E. coli* (EHEC) [34]. All of these invasion genes are associated with the characteristics of extraintestinal pathogenic *E. coli* (ExPEC), a major property of *E. coli* strains in Clermont type B2. In addition, SM47E1 (Clermont type B2) carries the highest number of predicted virulence genes (69 genes). These data show that SM47E1 is a food isolate with potential in disease etiology.

### 2.5. IncFIB (AP001918) Is the Most Frequently Detected Plasmid Replicon among E. coli Isolates in This Study

**Figure 5:**
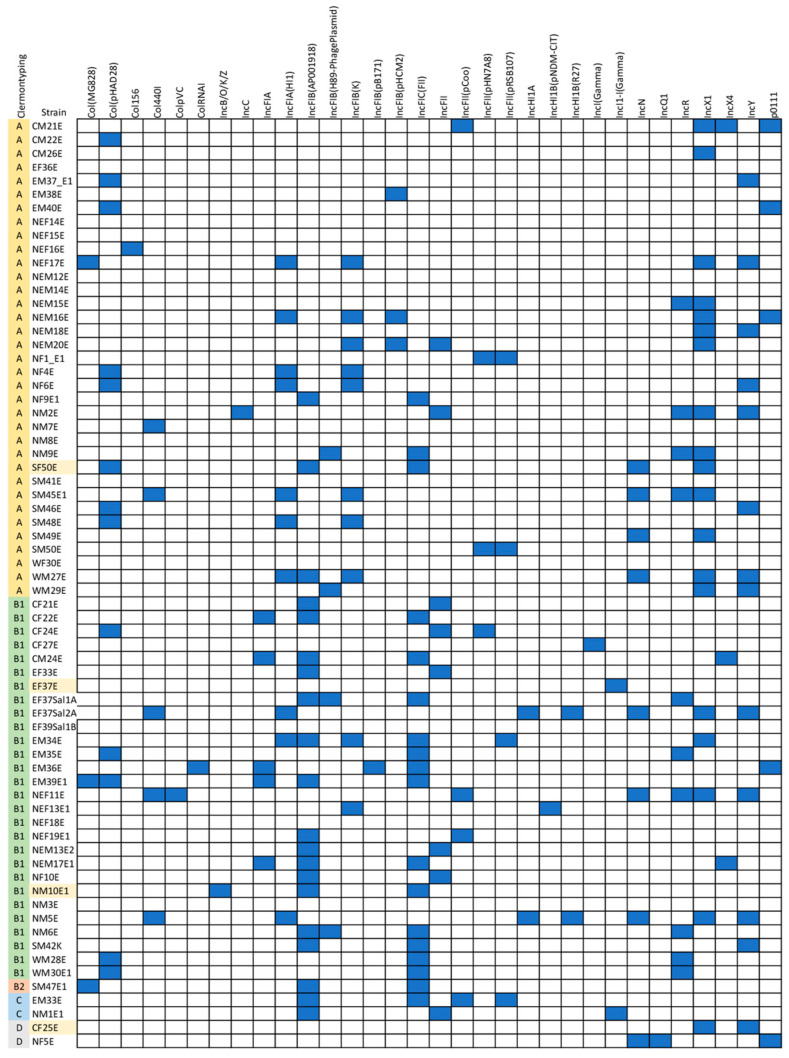
Histogram of plasmid replicon predictions from genomic data of *E. coli* isolates using PlasmidFinder. Blue boxes represent the presence of corresponding replicating plasmids.

Among the genomic data from 68 *E. coli* isolates, we identified 32 plasmid replicons, with the results shown in Figure 5 and the list of predicted plasmid replicons shown in Supplemental Table S6. We found 11 isolates (16.18%) without predicted plasmid replicons (EF36E, EF39Sal1B, NEF14E, NEF15E, NEF18E, NEM12E, NEM14E, NM3E, NM8E, SM41E, and WF30E). The most frequently predicted plasmid replicon was IncFIB (AP001918), found in 20 isolates (29.41%; CF21E, CF22E, CM24E, EF33E, EF37Sal1A, EM33E, EM34E, EM39E1, NEF19E1, NEM13E2, NEM17E1, NF10E, NF9E1, NM1E1, NM6E, NM10E1, SF50E, SM42E, SM47E1, and WM27E). The second and third most prevalent replicons were IncX1 (n = 19/68, 27.94%; CF25E, CM21E, CM26E, EF37Sal2A, EM34E, NEF11E, NEF17E, NEM15E, NEM16E, NEM18E, NEM20E, NM2E, NM5E, NM9E, SF50E, SM45E1, SM49E, WM27E, and WM29E) and IncFIC (FII) (n = 18/68, 26.47%; CF22E, CM24E, EF37Sal1A, EM33E, EM34E, EM35E, EM36E, EM39E1, NEM17E1, NF9E1, NM10E1, NM6E, NM9E, SF50E, SM42E, SM47E1, WM28E, and WM30E1), respectively. IncFIB (AP001918) was also the most frequently detected plasmid replicon in Clermont type B1 (n = 14/28, 50%); however, IncX1 was the most frequently detected plasmid replicon in Clermont type A (n = 14/35, 40%). Three isolates (EF37Sal2A, NEF11E, and NM5E) contained up to seven plasmid replicons, the highest number of plasmid replicons identified in the studied genome.

### 2.6. Positive Correlation between Gene Cluster for Yersiniabactin Production and Colistin Resistance Phenotype in Clermont Type B1

To analyze the correlation between genotype (AMR and virulence) and phenotype (AMR), the data on AMR patterns obtained from disk diffusion and microdilution (0 = sensitive, 1 = intermediate resistance or resistance) and genotypic data (0 = absence, 1 = presence) were analyzed using Pearson correlation. We found no correlations between phenotypes and genotypes among all strains of *E. coli* in this analysis (Supplemental Tables S7 and S8). Based on the patterns of AMR and virulence genes, each Clermont type appeared to have a specific pattern. Clermont type B2 contained one strain, whereas Clermont types C and D each contained two strains; thus, the numbers of *E. coli* strains from these three Clermont types (B2, C, and D) were not considered for correlation analysis. However, Clermont types A and B1 were reanalyzed for correlation between genotypes and phenotypes, and we found that only Clermont type B1 showed correlations between genotypes (AMR and virulence) and phenotypes (Table 2 and Supplemental Tables S9–S12).

In Clermont type B1, *floR* (phenicol resistance) showed a strong positive correlation with SXT resistance, whereas *dfrA14* (trimethoprim resistance) showed a strong positive correlation with COL resistance. Two categories of virulence genes, adherence and iron uptake, showed a strong positive correlation. The *afaBCDE-VIII* and *afa* operon (Afa pilus for adherence [38]) revealed a strong positive correlation, while *fimI* (adherence) showed a very strong negative correlation. Only two strains (CM24E and NEM17E1) in B1 carried the *afa* operon and lacked *fimI*. In the iron uptake category, *fyuA*, *irp12*, and *ybtAEPQSTUX* (*ybt* operon) showed strong positive correlations with COL resistance. The *fyuA*, *irp12*, and *ypt* operon are involved in the production of siderophore yersiniabactin in *Yersinia* spp. [39]. The gene cluster for producing yersiniabactin was also found in colistin-resistant high-risk clones of *Klebsiella pneumoniae* [40]. Iron acquisition by yersiniabactin might be associated with COL resistance.

## 3. Discussion

The presence of *E. coli* in food is among the persistent challenges in food sanitation and food safety. Specifically, multidrug-resistant and virulence factor-harboring *E. coli* in contaminated foods pose serious problems to human public health. There is a continuous need to monitor the incidence of AMR *E. coli* in the food chain. In this study, 68 *E. coli* strains were isolated from 72% of food samples (n = 72/100, 72%), including 44 (88%) from meat and 28 (56%) from fermented foods [15]. MDR strains were identified in 44.12% of all 68 strains, and 45.59% had MAR index values greater than 0.2. These AMR data reveal that the population of isolated *E. coli* from foods in Thailand presents a high risk of MDR incidence or exposure. The most prevalent resistance detected in our collection was AMP (64.71%), followed by TET (57.35%) and SXT (41.18%). AMP resistance was also detected in *E. coli* isolates (91%) from broilers, pigs, and their meat products from Sa Keao (a province in the eastern part of Thailand) [14]. Resistances to AMP, TET, and SXT are commonly found in *E. coli* strains isolated from humans or food-producing domestic animals [41]. Furthermore, we also identified COL-resistant (13.24%), IMI-resistant (1.47%), and ESBL-producing strains (10.29%), and our results are in accordance with increasing AMR incident reports in Thailand [11,14,42]. The high percentage of last-line drug resistance and ESBL producers is alarming and demonstrates the need for better hygiene control in the Thai food chain.

Based on whole genome sequencing, we found that the majority of isolated strains belong to Clermont type A (51.47%), followed by B1 (41.18%), C (2.94%), D (2.94%), and B2 (1.47%). Clermont types A, B1, and C are major clusters of commensal nonpathogenic *E. coli* [17]. However, we also found Clermont types B2 and D, which are mainly clusters of ExPEC. Although they were found in only three strains, we cannot disregard the potential of these isolates as foodborne *E. coli* to cause illness in consumers. Based on the Achtman scheme, we reported four new STs (CF25E, EF37E, NM10E1, and SF50E) from four different regions of Thailand. The new STs belong to Clermont types A (SF50E), B1 (EF37E and NM10E1), and D (CF25E). Therefore, the three new STs (SF50E, EF37E, and NM10E1) could be nonpathogenic or have low pathogenic potential, whereas CF25E could be an ExPEC.

Based on AMR gene prediction, we found that six strains harbored the *mcr* gene, and only two strains showed COL resistance (CM24E and NEM17E1). Four COL-resistant strains that lacked the *mcr* gene might have acquired other mechanisms for COL resistance. Interestingly, *mcr1* and *mcr3* were also found in *E. coli* isolates from pig, pig carcass, and pork products in Thailand, Laos PDR, and Cambodia [43]. The co-occurrence of *mcr1* and *mcr3* in colistin-susceptible *E. coli* was previously found in Thailand [43]. In our study, this co-occurrence was identified in SM42K, but the results indicated that *mcr1* and *mcr3* co-occurrence did not enhance colistin resistance [43]. The most prevalent *bla* is *bla*_TEM-1_, found in 31 strains (45.58%) from four Clermont types. In addition, *bla*_TEM-1_ was found in five ESBL-producing strains from a total of seven ESBL producers (71.43% of ESBL producers); hence, *bla*_TEM-1_ could be an important gene for ESBL production. Furthermore, we found the *bla*_EC_ gene group in all 68 strains, and some *bla*_EC_ genes were observed to be specific to a particular Clermont type; for example, *bla*_EC_ was found only in type A, while *bla*_EC-13_ was predominantly found in type B1. This pattern is similar to that of uropathogenic *E. coli* (UPEC) isolates from Saudi Arabia [44] and might be a predominant feature or specific to Clermontyping.

The *csgG* (adherence), *cheY* (chemotaxis), *ent* operon (iron uptake), *fep* operon (iron uptake), and *fes* (iron uptake) are virulence-associated genes that were found in all isolates and seem to be common among our strain collection. The virulence gene pattern also shows a correlation with Clermont types. Isolates in Clermont types A and B1 are common among commensal strains. The T3SS effector is found in Clermont type D, whereas the *chu* operon and genes for invasion are found in Clermont type B2, all of which are indicators of high pathogenic potential for ExPEC.

Regarding plasmid replicon prediction, we found more than 30 replicon types in 68 *E. coli* isolates. The results indicated that mobile genetic elements in *E. coli* isolates from food in Thailand have high genomic diversity. IncFIB (AP001918), the most frequently detected plasmid replicon among our *E. coli* isolates, was most frequently detected in *E. coli* from dairy animals [45,46]. Even though the relationships between detected plasmid replicons and phenotypic profiles are still unclear, we cannot disregard that plasmids and other mobile genetic elements can transfer AMR genes and/or virulence genes from a hypovirulent strain to a nonpathogenic strain, which can result in the emergence of a resistant and/or pathogenic strain [47,48]. The correlation between phenotype and genotype was explored using the antibiotic susceptibility test and whole genome sequencing. Overall, a correlation was present only in Clermont type B1. Clermont types B2, C, and D in our collection have one or a few strains; thus, the populations of those types were too low to identify specific correlation patterns. Further studies are still required to expand the data and analyze the relationships between genotypes and phenotype. Additionally, mutations and mobile genetic elements can affect antibiotic resistance activity and virulent features in *E. coli*. As this point cannot be underestimated, more extensive surveillance is needed to report the prevalence, MAR tracking, and high-risk virulent clones of *E. coli* in contaminated food.

## 4. Materials and Methods

### 4.1. Bacterial Strains and Cultivations

*E. coli* strains were isolated from raw and fermented meat in Thailand [15] using a conventional method that employed L-EMB and MacConkey agar as selective media, with confirmation via biochemical testing [49]. In this study, *E. coli* strains were cultured on Mueller–Hinton agar (MHA) (Difco^TM^, Leeuwarden, The Netherlands) and incubated at 37 °C overnight.

### 4.2. Antibiotic Resistance Susceptibility Testing

Antibiotic susceptibility was determined using the disk diffusion method for 11 drugs [amikacin (AMK, 30 μg, Oxoid^TM^, Basingstoke, UK), amoxicillin–clavulanic acid (AMC, 30 μg, Oxoid^TM^), ampicillin (AMP, 10 μg, Oxoid^TM^), cefotaxime (CTX, 30 μg, Oxoid^TM^), ceftazidime (CAZ, 30 μg, Oxoid^TM^), enrofloxacin (ENR, 5 μg, Oxoid^TM^), imipenem (IMI, 10 μg, Himedia^®^, Kennett Square, PA, USA), meropenem (MER, 10 μg, Himedia^®^), norfloxacin (NOR, 10 μg, Oxoid^TM^), tetracycline (TET, 30 μg, Oxoid^TM^), and sulfamethoxazole–trimethoprim (SXT, 25 μg, Oxoid^TM^)] and the microdilution method for colistin (COL, Sigma-Aldrich^®^, St. Louis, MO, USA). ESBL production was detected using the phenotypic confirmatory disk diffusion test (PCDDT) [50]. The zone of inhibitions was measured and the results were interpreted following the CLSI (Clinical and Laboratory Standard Institute) guidelines [50]. All strains which showed a diameter of inhibition zone less than 22 mm for ceftazidime and 27 mm for cefotaxime were selected to detect ESBL production. Combined antibiotic disks [clavulanic acid (10 μg)/CAZ (30 μg) and clavulanic acid (10 μg)/CTX (30 μg), BD BBL^TM^, Becton Drive Franklin Lakes, NJ, USA] and single antibiotic disks [CAZ (30 μg) and CTX (30 μg), BD BBL^TM^] were used. A greater than 5 mm increase in the inhibition zone for the combined antibiotic disk versus the single antibiotic disk confirmed ESBL-producing strains. For the microdilution method, cell suspensions were diluted in CAMHB (Cation-adjusted Mueller–Hinton Broth, Difco^TM^) to 1 × 10^6^ CFU/mL. The 2-fold serial dilutions of colistin were prepared and mixed with 5 × 10^5^ CFU/mL *E. coli* in a total volume of 200 μL. Minimum inhibitory concentration (MIC) was observed and cell viability was measured with MTT-based staining [51]. Colistin resistance was determined according to the CLSI guidelines (MIC ≥ 4 μg/mL; resistant) [50]. *E. coli* ATCC 25922 was used as a quality control strain for the disk diffusion and microdilution methods. The multiple antibiotic resistance (MAR) index was calculated by dividing the number of antibiotics to which an isolate was resistant by the total number of antibiotics tested [52].

### 4.3. Genomic DNA Extraction and Whole Genome Sequencing

Whole genomic DNA was extracted using a modified Marmur procedure [53]. Briefly, bacterial cells were harvested from a 3 mL culture in CAMHB and resuspended in EDTA-saline (0.01 M EDTA and 0.15 M NaCl, pH 8.0). Subsequently, 30 μL 110 mg/mL lysozyme and 10 μL 20 mg/mL RNase A were added and incubated at 37 °C for 2 h. After incubation, 80 μL 20% SDS and 10 μL 5 mg/mL proteinase K were added and incubated again at 65 °C for 30 min. Thereafter, 5 M NaCl was added at a volume of 0.5, followed by phenol–chloroform extraction. The upper liquid phase was transferred to a new tube, and volumes of 0.25 of 5 M NaCl and 0.1 of 3 M sodium acetate were added. Ice-cold absolute ethanol was added at 2 volumes and inverted gently. A DNA pellet was hooked and transferred into a new microcentrifuge tube, air-dried, and resuspended in DNase- and RNase-free water. The quality and quantity of DNA were measured using a UV spectrophotometer, Qubit dsDNA BR assay kit (Invitrogen^TM^, Waltham, MA, USA), and 1% agarose gel electrophoresis. Subsequently, 300 ng extracted DNA was used for library preparation using the MGIEasy FS DNA Library Prep Set, followed by paired-end sequencing on an MGI sequencer at the National Science and Technology Development Agency (NSTDA).

### 4.4. Genome Analysis

Raw read quality was assessed using FastQC version 0.11.9 [54]. The quality of raw reads were measured using MegaBOLT version 1.5.6.11; and the reads were assembled using Unicycler version 0.4.8 [55]. The quality of assembled contigs were assessed based on 3C criterion [56] in the Galaxy server [57], using QUAST version 5.2.0 [58] to evaluate contiguity and correctness (compared to complete chromosomal genome of *E. coli* ATCC 25922, accession number: CP009072.1), and compleasm version 0.2.6 [59] and CheckM-genome version 1.2.3 [60] to assess completeness and contamination. De novo assembled contigs showed that the total base of the genome was approximately 4.5–5 Mbp. Genome coverages were greater than 30×, most contigs were larger than 1000 bp, and completeness analysis showed that more than 95% were considered for analysis. De novo assembled contigs were used for the identification of ribosomal sequence types (rSTs) using rMLST (ribosomal multi-locus sequence typing) and of STs using MLST according to the Achtman scheme (based on the *adk*, *fumC*, *gyrB*, *icd*, *mdh*, *purA*, and *recA* genes) [61] in pubMLST version 1.42.6 [62] and Enterobase version 1.2.0 [63]. The O and H antigens of *E. coli* were predicted using SerotypeFinder version 2.0 in the CGE (Center of Genomic Epidemiology) server [64] and Enterobase version 1.2.0 [63]. Coding sequences and functional genes were annotated using BV-BRC version 3.30.19 [65]. Lineage phylogroups and Clermontyping were determined using Enterobase version 1.2.0 [63]. Antibiotic resistance genes were predicted using the ResFinder version 4.1 [66] database and the National Database of Antibiotic-resistant Organisms (NDARO) [67], and virulence factor genes were predicted using the VFDB database [68]. Plasmid replicons were predicted using PlasmidFinder [69]. With identity and gene coverage equal to or greater than 90%, and an E-value of less than 0.01, the genes were predicted as antibiotic resistance and virulence genes. The core genome phylogenetic tree was constructed using BV-BRC version 3.30.19, with 1000 core genes (Supplemental Table S13) for analysis [65], and visualized using iTOL version 5 [70]. The whole genomic sequence of *E. fergusonii* strain NCTC 12128 (accession number: UGFW00000000.1) was used as an outgroup for core genome phylogenetic analysis.

### 4.5. Correlation and Statistical Analysis

The correlation between genotype and phenotype was analyzed using Pearson’s correlation coefficient [71]. The correlation coefficient values were interpreted as follows: 0.70 to 0.90 indicated strong positive correlation, 0.90 to 1.00 indicated very strong positive correlation, −0.70 to −0.90 indicated strong negative correlation, and −0.90 to −1.00 indicated very strong negative correlation. A correlation between two groups was considered statistically significant if the *p*-value was less than 0.05.

## 5. Conclusions

Among the 68 strains of *E. coli* investigated in this study, 31 (or 45.59%) were MDR isolates, of which seven were resistant to last-line drugs, such as colistin. We also identified four isolates with new STs, according to the Achtman scheme sequence types. Even though most isolates did not show high potential as pathogens based on their virulence gene profiles, we should not underestimate the prevalence of AMR genes in foods. This study demonstrates the pressing problem of antimicrobial resistance and virulence genes being transmitted among bacteria found in the food matrix, particularly in Thailand. Understanding the correlations between genotypic and phenotypic information provides insights into food safety surveillance and contamination control.

## Figures and Tables

**Table 1:** AMR patterns and MAR index values of *E. coli* isolates.

AMR Pattern ^A^	Number of Strains	% Population	MAR Index
AMC-AMP-CAZ-COL-CTX-ENR-IMI-NOR-TET-SXT	1	1.47	0.83
AMC-AMP-CAZ-COL-CTX-ENR-TET-SXT	1	1.47	0.67
AMC-AMP-CAZ-CTX-ENR-NOR-TET-SXT	1	1.47	0.67
AMC-AMP-CAZ-CTX-TET-SXT	1	1.47	0.50
AMC-AMP-COL-ENR-TET-SXT	1	1.47	0.50
AMP-CAZ-CTX-ENR-NOR-TET	1	1.47	0.50
AMP-COL-ENR-TET-SXT	2	2.94	0.42
AMP-CTX-ENR-TET-SXT	1	1.47	0.42
AMC-AMP-ENR-TET	1	1.47	0.33
AMC-AMP-TET-SXT	1	1.47	0.33
AMP-CAZ-CTX-ENR	2	2.94	0.33
AMP-COL-TET-SXT	1	1.47	0.33
AMP-ENR-TET-SXT	3	4.41	0.33
AMC-AMP-SXT	1	1.47	0.25
AMP-ENR-SXT	1	1.47	0.25
AMP-ENR-TET	1	1.47	0.25
AMP-TET-SXT	11	16.18	0.25
AMC-AMP	1	1.47	0.17
AMP-CTX	1	1.47	0.17
AMP-TET	9	13.24	0.17
TET-SXT	2	2.94	0.17
AMP	2	2.94	0.08
TET	1	1.47	0.08
ND (Not detected; sensitive for all selected antibiotics)	21	30.88	0.00

^A^ Abbreviations: AMC = amoxicillin–clavulanic acid; AMK = amikacin; AMP = ampicillin; CTX = cefotaxime; CAZ = ceftazidime; COL = colistin; ENR = enrofloxacin; IMI = imipenem; MER = meropenem; NOR = norfloxacin; TET = tetracycline, SXT = sulfamethoxazole–trimethoprim.

**Table 2:** List of statistically significant strong/very strong correlations between genotypes and phenotypes.

Comparison	Correlation Coefficient	*p*-Value	Interpretation
AMR genotype vs. AMR phenotype (Clermont type B1)			
*floR* (phenicol resistance) vs. SXT	0.759	2.87 × 10^−6^	strong positive correlation
*dfrA14* (trimethoprim resistance) vs. COL	0.801	3.12 × 10^−7^	strong positive correlation
Virulence genotype vs. AMR phenotype (Clermont type B1)			
*afaBCDE-VIII* (adherence) vs. COL	0.801	3.12 × 10^−7^	strong positive correlation
*fimI* (adherence) vs. COL	−1.000	4.9 × 10^−205^	very strong negative correlation
*fyuA* (iron uptake) vs. COL	0.849	1.17 × 10^−8^	strong positive correlation
*irp12* (iron uptake) vs. COL	0.849	1.17 × 10^−8^	strong positive correlation
*ybtAEPQSTUX* (iron uptake) vs. COL	0.849	1.17 × 10^−8^	strong positive correlation

COL = colistin resistance phenotype, SXT = sulfamethoxazole–trimethoprim resistance phenotype.

## Data Availability

All genomic data have been deposited into DDBJ/ENA/GenBank under the BioProject ID PRJNA717915 “https://www.ncbi.nlm.nih.gov/bioproject/PRJNA717915” (accessed on 12 March 2024) and Enterobase (Supplemental Table S14).

## Supplementary Materials

The following supporting information can be downloaded at https://www.mdpi.com/article/10.3390/antibiotics13100935/s1, Supplemental Table S1: AMR phenotypic patterns and MAR index values; Supplemental Table S2: Data of quality assessment for assembled genome sequences; Supplemental Table S3: List of *E. coli* strains with identification and source data; Supplemental Table S4: List of AMR genes; Supplemental Table S5: List of virulence genes; Supplemental Table S6: List of plasmid replicons; Supplemental Table S7: Correlation matrix of AMR genes and AMR phenotypes in all *E. coli* isolates; Supplemental Table S8: Correlation matrix of virulence genes and AMR phenotypes in all *E. coli* isolates; Supplemental Table S9: Correlation matrix of AMR genes and AMR phenotypes in Clermont type A; Supplemental Table S10: Correlation matrix of AMR genes and AMR phenotypes in Clermont type B1; Supplemental Table S11: Correlation matrix of virulence genes and AMR phenotypes in Clermont type A; Supplemental Table S12: Correlation matrix of virulence genes and AMR phenotypes in Clermont type B1; Supplemental Table S13: List of core genes for constructing core genome phylogenetic tree; Supplemental Table S14: List of Enterobase barcodes and accession numbers from NCBI for *E. coli* isolates.
